# Chemotherapy and skin reactions

**DOI:** 10.1186/1756-9966-31-50

**Published:** 2012-05-28

**Authors:** Gabriella Fabbrocini, Norma Cameli, Maria Concetta Romano, Maria Mariano, Luigia Panariello, Dario Bianca, Giuseppe Monfrecola

**Affiliations:** 1 Division of Clinical Dermatology, Department of Systematic Pathology, University of Naples Federico II, Via Sergio Pansini 5, 80133, Naples, Italy; 2 San Gallicano Dermatological Institute, IRCCS, Via Elio Chianesi 53, 00144, Rome, Italy; 3 ASL ROMA C - School of University Tor Vergata, Rome, Italy

**Keywords:** Chemotherapy, Skin toxicity, Follicular rash, Anti-EGF, Xerosis, Paronychia

## Abstract

**Background:**

New chemotherapic agents and new protocols in oncology have led to an increasing survival rate in patients affected by tumors. However, this increased use has been accompanied by a growth in the incidence of cutaneous side effects and a worsening of patients’ quality of life. Appropriate management of skin toxicity associated with chemotherapic agents is therefore necessary for suitable drug administration and to improve quality of life and clinical outcomes.

**Methods:**

We have clinically examined 100 patients affected by cancer, determining type, frequency, treatment, and evolution of side effects related to chemotherapy.

**Results:**

The prevalent cutaneous side effects in patients undergoing chemotherapy are skin rash, xerosis, pruritus, paronychia, hair abnormality, and mucositis. The clinical cases are reported in detail.

**Conclusion:**

Oncological therapies have become more selective and have low systemic toxicity because of their high specificity, but cutaneous side effects are common and may worsen the quality of life of these patients.

## Background

Over the last two decades, a number of new chemotherapeutic agents have been used for the treatment of cancer.

These drugs may be classified according to their mechanism of action in: *Signal transduction inhibitors* (Epidermal growth factor receptor – EGFR- antagonists and Multikinase inhibitors), *Proteasome inhibitors, Spindle inhibitors* (Taxanes and Vinca alkaloids), *Antimetabolites* (Purine analogs and Pyrimidine analogs), *Genotoxic agents*[1]*.*

Chemotherapeutic agents have significant side effects. Although skin toxicity is rarely life-threatening it often worsens the patients’ quality of life.

It is well known that, cytotoxic agents like Cyclophosphamide, Chlorambucil, Busulfan, Procarbazine can cause side-effects on hair and nails (alopecia, paronychia, melanonychia, and other abnormalities), on skin barrier (skin rash, skin dryness, hyperpigmentation) and on mucose (Steven-Johnson Syndrome and toxic epidermic necrolysis).

In recent years, targeted therapy has considerably increased survival rate in patients affected by important solid tumors of kidney, lungs, colon-rectum, breast and liver. Among the innovative therapeutic strategies in chemotherapy, the EGFR inhibitors (Cetuximab, Panitumumab, Erlotinib, Gefitinib) approved for lung and colon-rectum tumors showed an increasing skin toxicity, causing widespread skin dryness (in more than 90% of patients) and a follicular rash which can be complicated by pruritus, pain and infections [2,3]

Despite the benefits of all these chemotherapic agents, toxic effects on the skin may eventually result in poor compliance of patients and interruptions or discontinuation of antineoplastic therapy [4,5]. Such toxic effects of the skin may also significantly reduce the quality of life of oncological patients .

The aim of our study is to present all cases of mucocutaneous side effect of these new drugs referring to 3 outpatient departments for the skin care of oncological patients in Naples and Rome from October 2010 through December 2011.

## Methods

From October 2010, 3 outpatient departments for the skin care of oncological patients have been set up: the Department of Dermatology at the University Federico II in Naples, the San Gallicano Dermatological Institute and the ASL Roma C in Rome. We have collected data from the outpatient departments of these Dermatological Units of 100 patients in chemo and radiotherapy (35 males and 65 females), aged from 24 to 80 years (mean age 58 ± 7,5). We included in the study patients in chemotherapy of both sex, suffering from mucocutaneous side effects which had begun after the first administration of the drug. We excluded patients under radiotherapy and patients in which mucocutaneous symptoms were already present at the beginning of chemotherapy. Every side effect has been evaluated by Common Terminology Criteria for Adverse Events (CTCAE) version 4.03 [6]. The patients’ data has been registered using a software set up specifically to record the patients’ general information, tumor grading, type of chemotherapy. Moreover skin of patients affected by dry skin and skin rashes was instrumentally evaluated by corneometry, trans-epidermal water loss (TEWL) and colorimetry.

Corneometry evaluation has been performed using the Corneometer CM 820 PC Courage (Courage + Khazaka electronic Mathias-Brüggen-Str. 91 D-50829 Köln (Germany)), which measures skin conductance through low intensity electric current. This value is inversely related to skin water content of the stratum corneum and gives a direct measurement of skin hydration units.

The Tewameter device (Tewameter TM 210 Courage – Khazaka electronic) measures the amount of transepidermal water loss (TEWL) and has been used to determine skin hydration grade with moisture and temperature sensors.

Colorimetry analysis has been performed by Spectrocolorimeter (X-Rite), whose special probe makes it possible to evaluate skin color according to the L* a* b* parameters. We have considered only the L* value, which represents the relative brightness between total black and total white.

Different dermocosmetical therapies were performed on the basis of different mucocutaneous reactions. Patients were observed at time 0 (first visit) and time 30 (after 30 days). We also performed *χ*^2^ square test to compare different adverse drug reactions and type of drugs administred.

This study has been performed with the approval of the Institutional Review Board of Department of Dermatology, University of Naples “Federico II”. It is in compliance with the Helsinki Declaration.

## Results

Samples were collected from 100 patients affected by: breast cancer (45 patients), colon-rectal cancer (10 patients), lung cancer (10 patients), prostate (4 patients), Hodgkin’s lymphoma HL (4 patients), stomach cancer (4 patients), thyroid cancer (4 patients), leukaemia (3 patients), Non-Hodgkin lymphomas NHL (3 patients), ovary cancer (2 patients), uterus cancer (2 patients), liver cancer (2 patients), kidney cancer (2 patients), oesophagus cancer (2 patients), neuroendocrine cancer (2 patients), schwannoma (1 patient).

Thirty-four of our patients were under treatment with EGFR-inhibitors and in particular with Cetuximab, Erlotinib, Lapatinib, Gefitinib and Panitumumab; 23 patients underwent hormonal therapy; 17 patients were in therapy with traditional drugs (i.e. Genotoxic agents Spindle inhibitors, Antimetabolites).

In the EGFR-inhibitors group we observed 19 papulo-pustular reactions (55.88% of patients). 14 patients showed dry skin (41.17%) and 10 nail alterations (29.41%). Only 6 patients (17.64%) suffered from hair alteration including alopecia and anagen effluvium (Additional files 1 and 2).

Patients under hormonal therapy mostly suffered from dry skin (14 patients, 60.86%). In this group we also observed hair alterations (5 patients, 21.73%) and nail alterations (6 patients, 26.08%) (Additional file 2 and 3).

Patients who had assumed traditional drugs showed dry skin (10 patients, 58.82%) and hair and nail alterations (6 and 4 patients respectively, 35.29% and 23.59%) (Additional file 2 and 4).

The *χ*^2^ square test we performed to evaluate different EGFR-inhibitor molecules showed a higher prevalence of follicular reactions induced by antibodies (Cetuximab and Panitunumab) in comparison with small molecules (Erlotinib, Gefitinib and Lapatinib) p <0,005. Occurrence of xerosis instead was higher with hormonal therapy than with EGFR-inhibitors p < 0.005.

**Figure 1:**
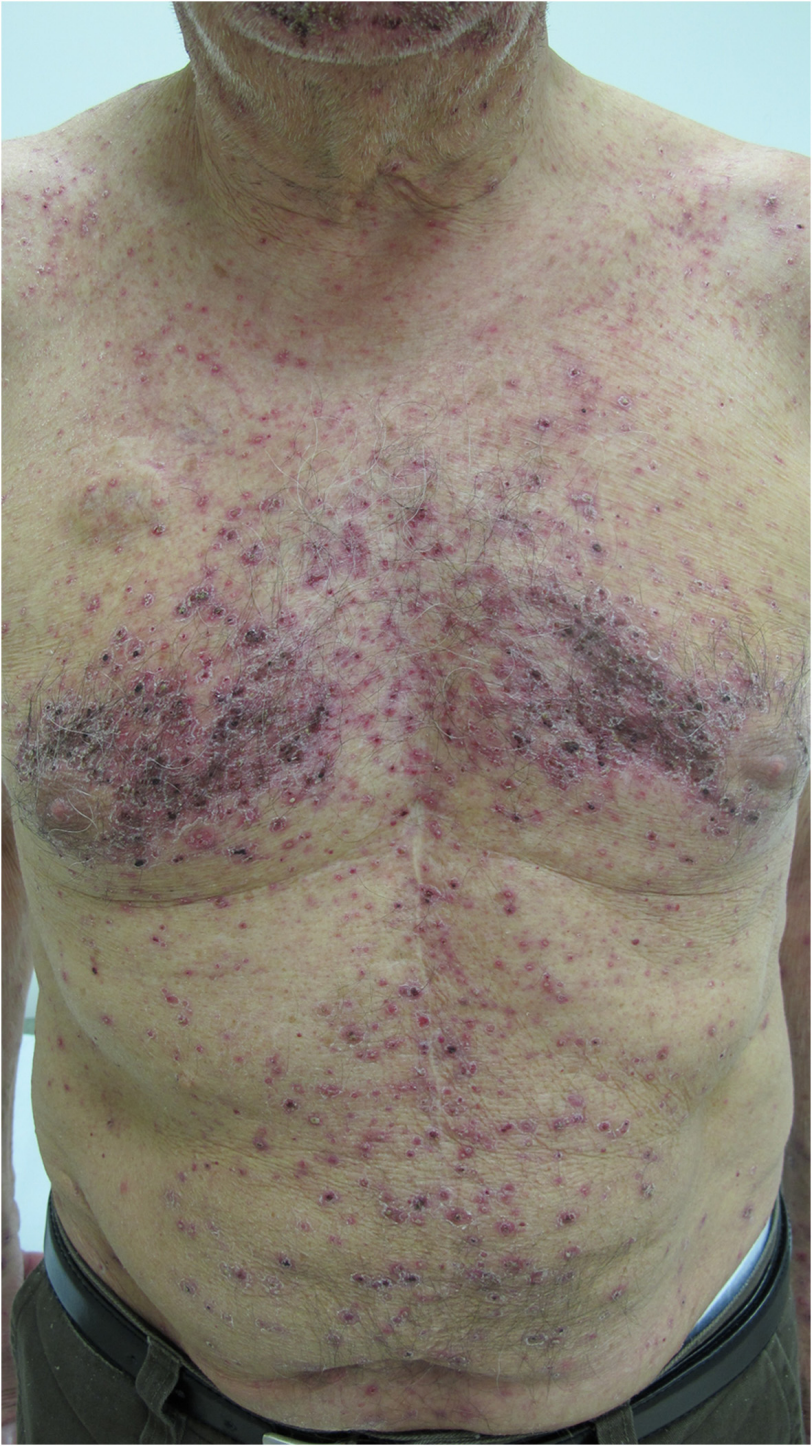
Panitunumab-related follicular rash.

**Figure 2:**
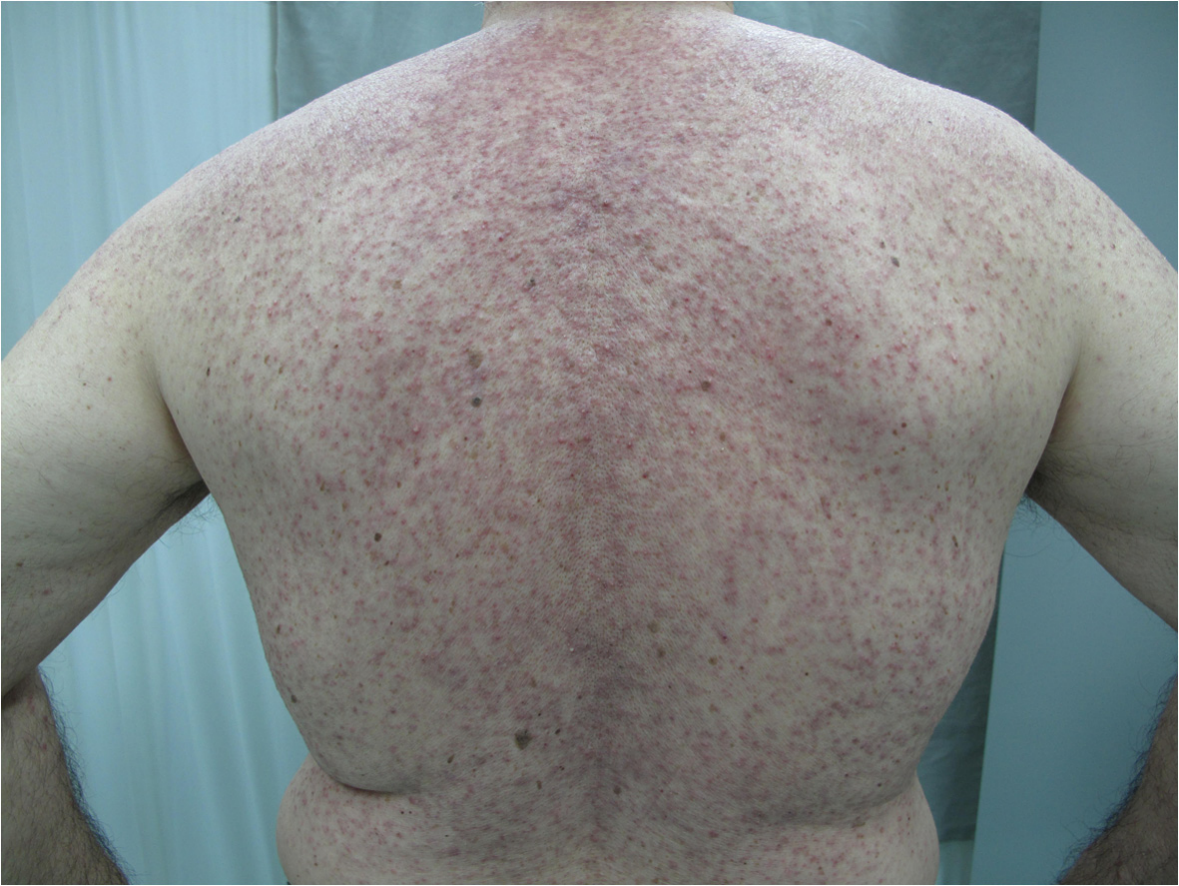
Follicular rash induced by Cetuximab.

In accordance with the current literature the follicular rash (Figures 1 and 2) usually occurred a few days after administration of the drug and reached a maximum after 2–3 weeks. The skin lesions consist of erythematous follicular papules that may evolve into pustules, localized on the face, neck and retroauricular area, scalp and upper trunk.

**Figure 3:**
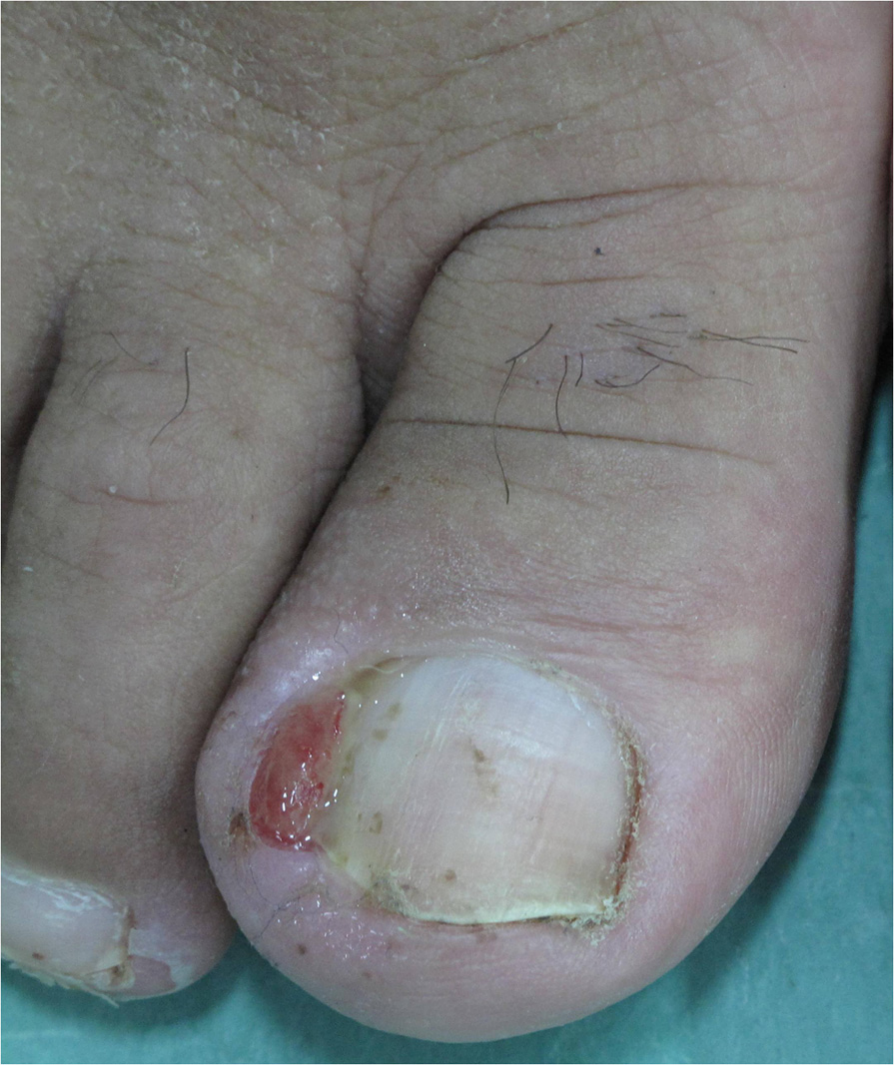
Paronychia in a female patient treated with Lapatinib.

**Figure 4:**
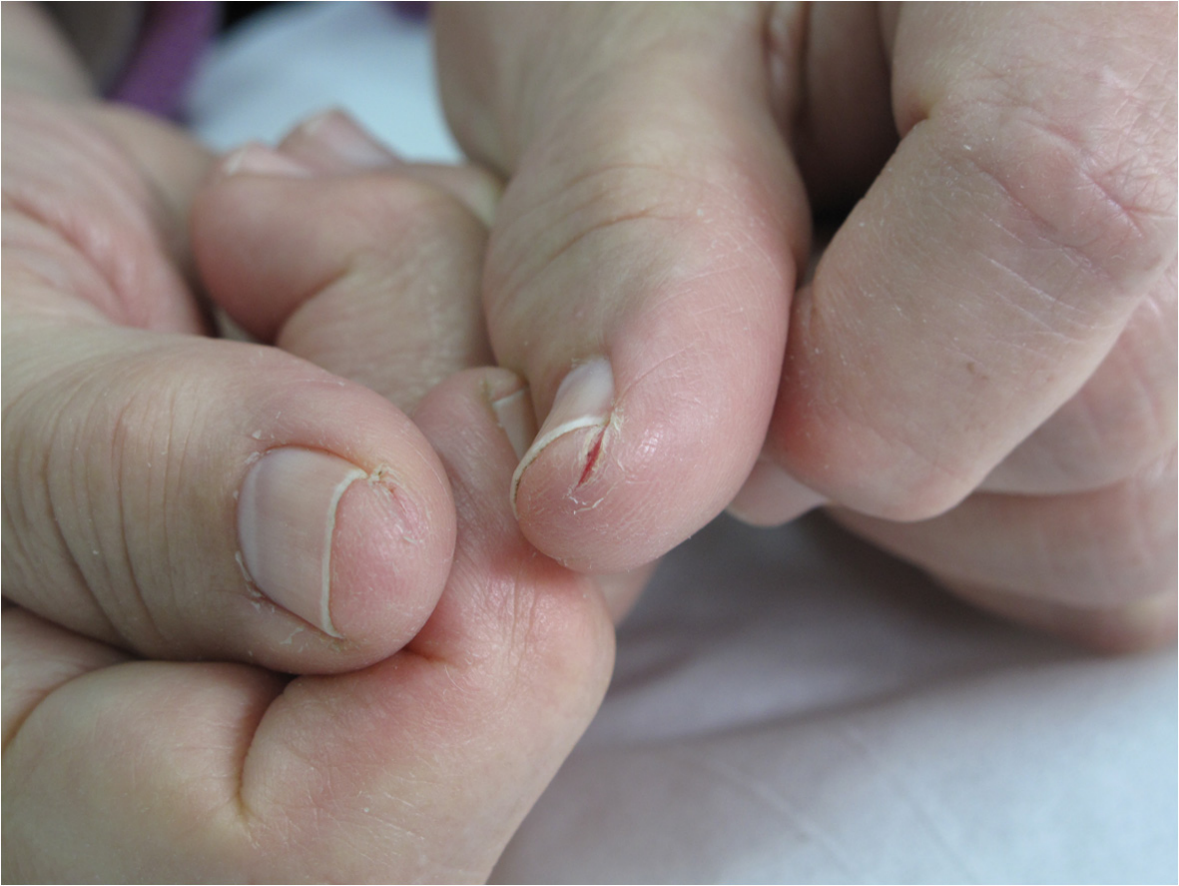
Fissures of the fingertips in a patient treated with taxanes.

Nail alterations, consisting mostly in frailer nails and paronychia (Figure 3) were often associated with painful fissures of the fingertips (Figure 4).

All the patients with xerosis and skin rashes were instrumentally evaluated by Corneometer, Tewameter and Spectrocolorimeter to study the correlation between such cutaneous toxicities and skin hydration, skin barrier function and skin brightness at the baseline and during cutaneous therapy. Corneometry examination showed average values between 0 and 50 in all the patients examined, which indicated high skin dehydration at the baseline (T0). A high majority of subjects also had signs of skin barrier function damage indicated by the Tewameter measurement (average values: 16.67 g/m^2^h) and low brightness values (L*). The dermatologic therapy suggested to these patients improved in all cases the Corneometer and Tewameter value.

## Discussion

Signal transduction inhibitors, in particular EGFR-antagonists, are a new class of chemotherapic agents, whose side effects result to be in dermatologic clinical practice [4,5].

The most frequently reported toxic cutaneous effect deriving from these drugs is the papulo-pustular follicular rash, which is defined as a form of acne since it involves above all the face’s seborrhoeic areas, scalp and chest and less frequently the extremities and the back. Such an eruption appears during the first two weeks of treatment [2,3], accompanied by an extremely irritating pruritus and can be complicated by bacterial over-infections, albeit short-lived. Its peculiar characteristic is the association of a typical sebaceous gland disease with a marked xerosis, indicating that the main pathogenetic factor is not the cutaneous adnexa but the keratinocyte itself.

The EGFR receptor is expressed in the basal layer of the epidermis and promotes the differentiation of keratinocytes and follicular cells. Moreover, EGFR-inhibitors inhibit not only the EGFR when overexpressed in tumor cells, but also the receptor present on normal cells of the epidermis. The inhibition of EGFR in normal skin leads to alterations of growth and migration of keratinocytes that, together with inflammatory reactions, lead to xerosis and papulopustolar skin rash. Mucosa and cutis xerosis, varying from light to more severe forms with eczema and fissures, has so far shown a variable incidence from 12% to 35% in clinical trials [7,8] and it often represents one of the cutaneous parameters persistently influencing the patient’s quality of life.

Nail alterations are frequently connected to the use of EGFR-inhibitors. The pathogenesis is unknown but it might be related to increased skin fragility induced by the treatment [2]. The clinical manifestation may be paronychia or periungual abscesses, which are usually a late sign of toxicity with an onset of about two months from beginning of the therapy. The first lesions are usually localized on the big toe. The toes present a very painful erythema.

Antimetabolites, 5-FU and Capecitabine in particular, result in a distinctive sign of toxicity: hand-foot syndrome, more frequent with Capecitabine. Patients can show erythema and swelling in mild cases, or in severe cases, blisters ulceration and desquamation. Patients also refer numbness and paraesthesia. Lesions are located on the palms of hands and soles of the feet. Another sign of skin toxicity linked to the use of Capecitabine is hyperpigmentation. This abnormality is also observed with Cyclophosphamide and Doxorubicin [9-12]. Patients can present black longitudinal pigmentation of the nails without any symptoms. These drugs are also connected to focal skin pigmentation, mainly involving the fingertips, combined with paresthesia or pain. According to some authors these manifestations may be considered as initial signs of the hand-foot syndrome [10]. The exact pathogenesis is unknown but it may be related to the increased expression in the skin of the fingertips of the enzymes necessary for Capecitabine activation in 5-FU. Damage of the nerve fibres seems to be the cause of the neuropathic symptoms [10].

Spindle inhibitors, i.e. Vinca alkaloids and Taxanes, approved for many solid and hematological tumors, are strictly related to alopecia and other skin diseases such as dermatitis, radiation recall, subacute cutaneous lupus erythematosus, nail abnormalities and ulcerations caused by extravasation, this reaction in particular is caused by direct damage to soft tissue.

Genotoxic agents may cause severe, well-known, allergic IgE-mediated reactions, in particular Platinum agents but also antibiotics. These can also lead to alopecia, because of their targeting on proliferating cells, and particular effects like erythema flagellatum whose pathogenesis is unknown.

Multikinase inhibitors used in hematology like Imatinib, Dasatinib and Nilotinib seem to be connected to frequent skin toxicity mainly consisting of dermatitis, sometimes exfoliative, associated with fever [1] and frequently with edema. Sorafenib and Sunitinib are two other multikinase inhibitors used for kidney and liver cancer. Inflammatory actinic keratosis has also been observed [13,14].

Sunitinib is associated to bullous manifestation and hand-foot syndrome, which can also be used as a marker of drug efficacy [15].

## Conclusions

New drugs and new therapeutic schedules have brought many malignancies to a better prognosis and a longer survival. However newer drugs, in particular targeted therapies, often provoke side effects on the skin, obliging physicians to suspend therapy. For this reason the challenge of future studies in this field is to identify methods capable to prevent this kind of side effects and, at the same time, specific therapies for each skin problem. Cooperation between oncologists and dermatologists is also fundamental in order to make the best decisions for the patients and to implement preventive measures.

## Abbreviation

EGFR, Epidermal growth factor receptor; i.e., Id est (that is); ASL, Azienda sanitaria locale (Local Health); TEWL, Trans epidermal water loss; HL, Hodgkin lynphoma; NHL, Non-Hodgkin lynphoma; ABVD, Adriamycine bleomycine vincristine dacarbazine; 5FU, 5-fluorouracile.

## Competing interest

The authors have no competing interest to declare. The manuscript is not under simultaneous consideration by any other publication. The content in this format had not been published yet.

## Authors’ contribution

GF designed the study and participated in its coordination. MCR carried out clinical evaluation of patients. NC administrated the best therapy for each patient in accordance with international literature and guidelines. MM recorded information about each patient and monitored their response to therapy. GP and DB participated in data processing. GM participated as supervisor of the study. All authors read and approved the final manuscript.

## Supplementary Material

Additional file 1 EGFR-inhibitors skin toxicities. Click here for file

Additional file 2 Compared frequency of skin adverse reactions among different group of drugs. Click here for file

Additional file 3 Hormonal therapy skin adverse reactions. Click here for file

Additional file 4 Traditional drugs skin toxicities. Click here for file
