# Supplementary figures and images for: Chemotherapy and skin reactions
Source: J Exp Clin Cancer Res. 2012 May 28;31(1):50. doi: 10.1186/1756-9966-31-50 (PMC3583303; doi:10.1186/1756-9966-31-50)

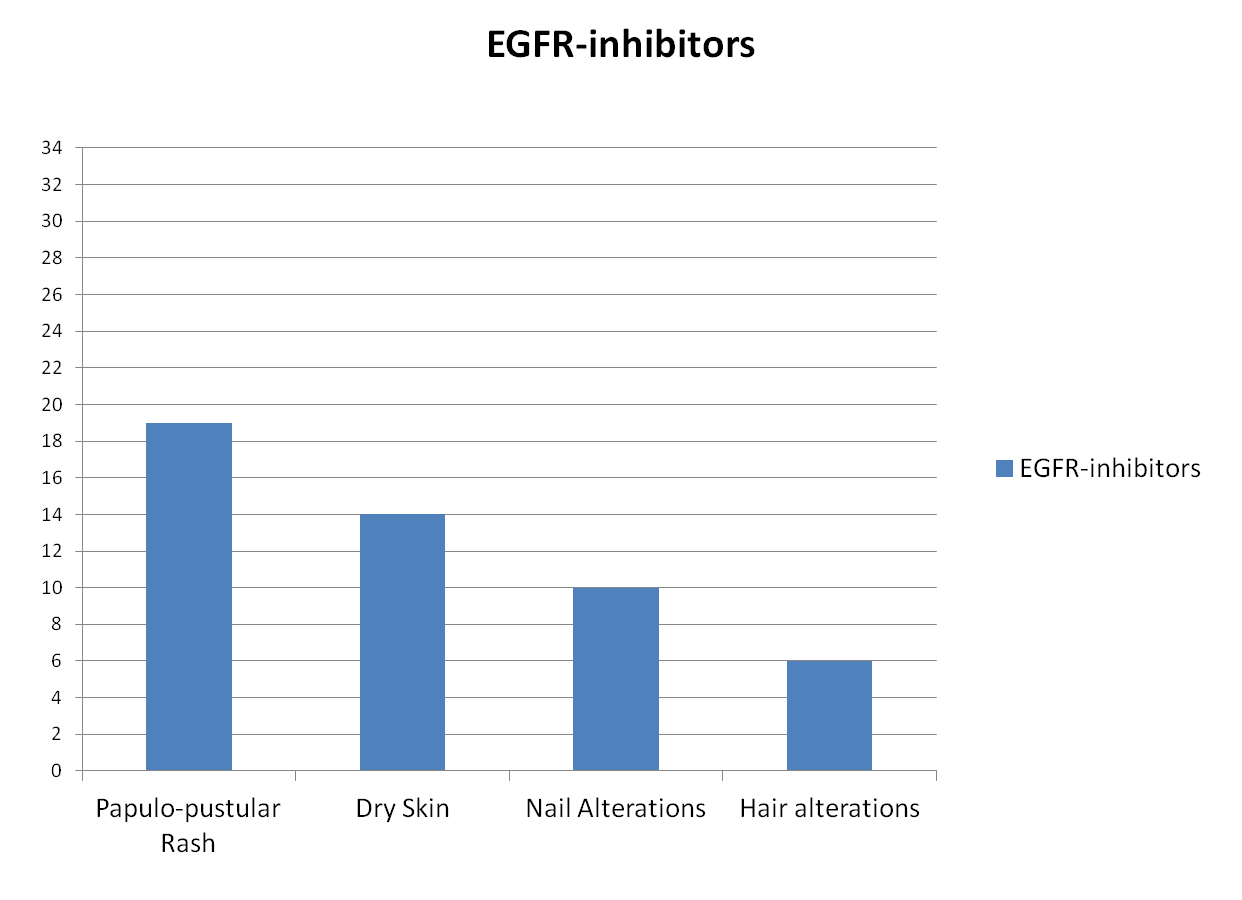

Supplement: Additional file 1 — EGFR-inhibitors skin toxicities. [file 1756-9966-31-50-S1.png]

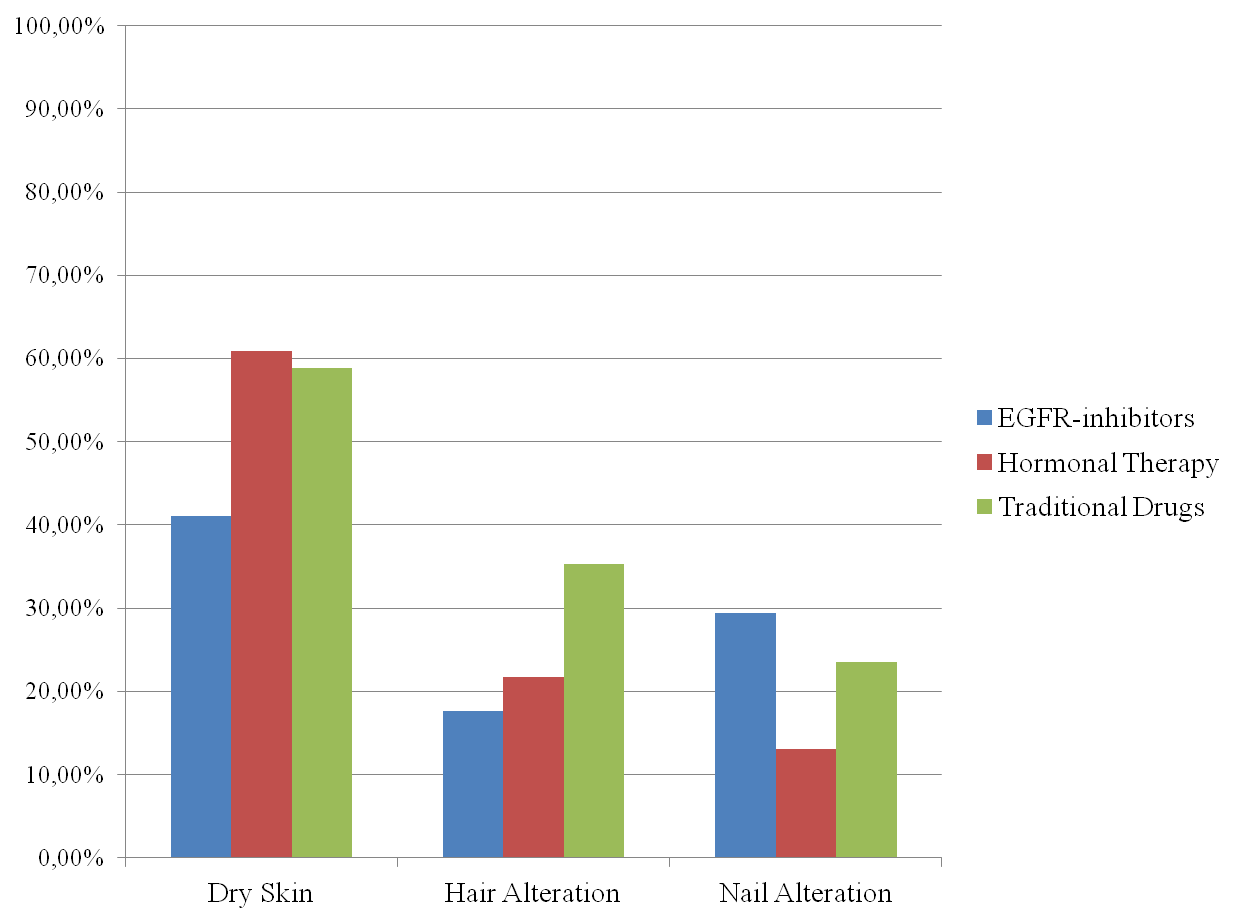

Supplement: Additional file 2 — Compared frequency of skin adverse reactions among different group of drugs. [file 1756-9966-31-50-S2.png]

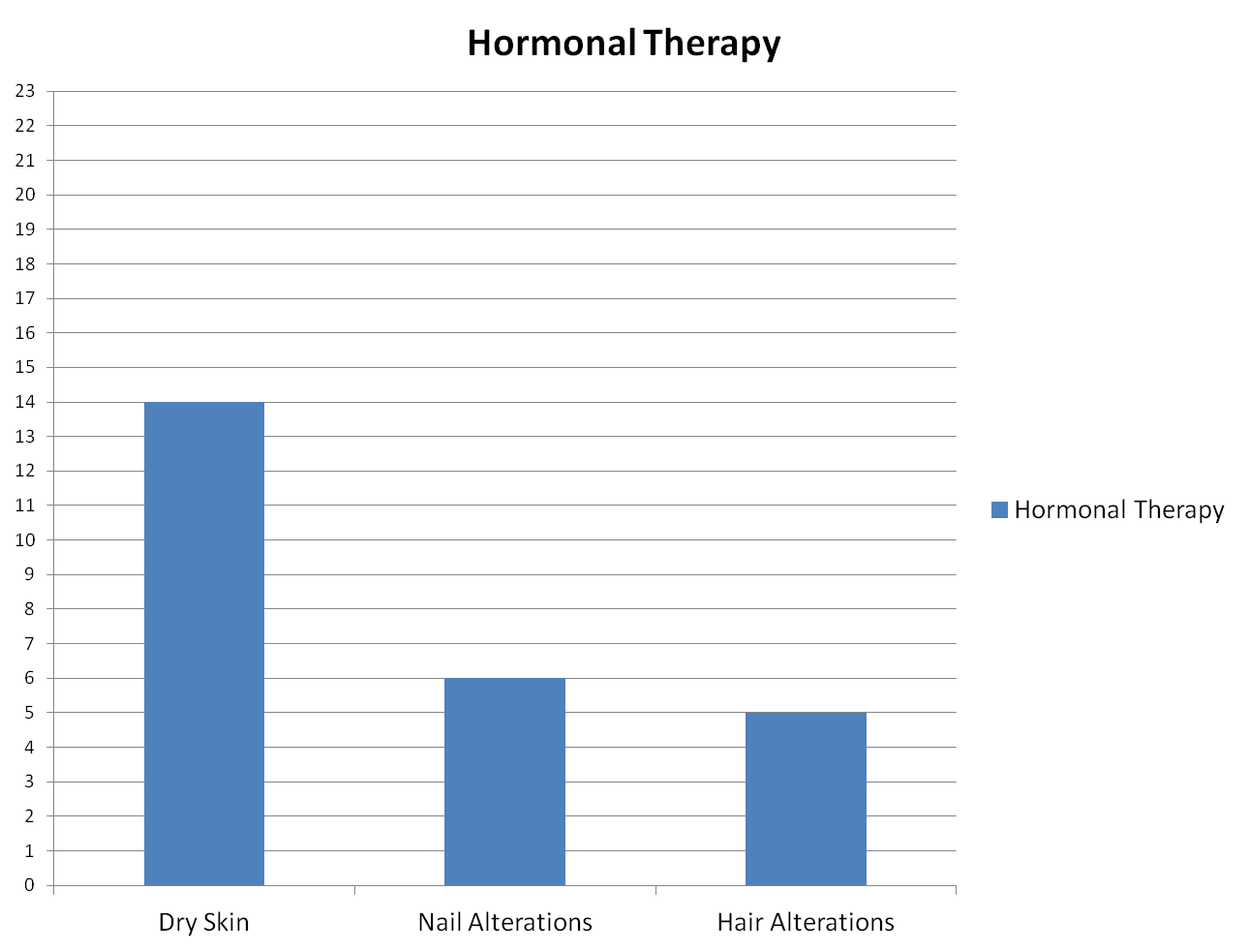

Supplement: Additional file 3 — Hormonal therapy skin adverse reactions. [file 1756-9966-31-50-S3.png]

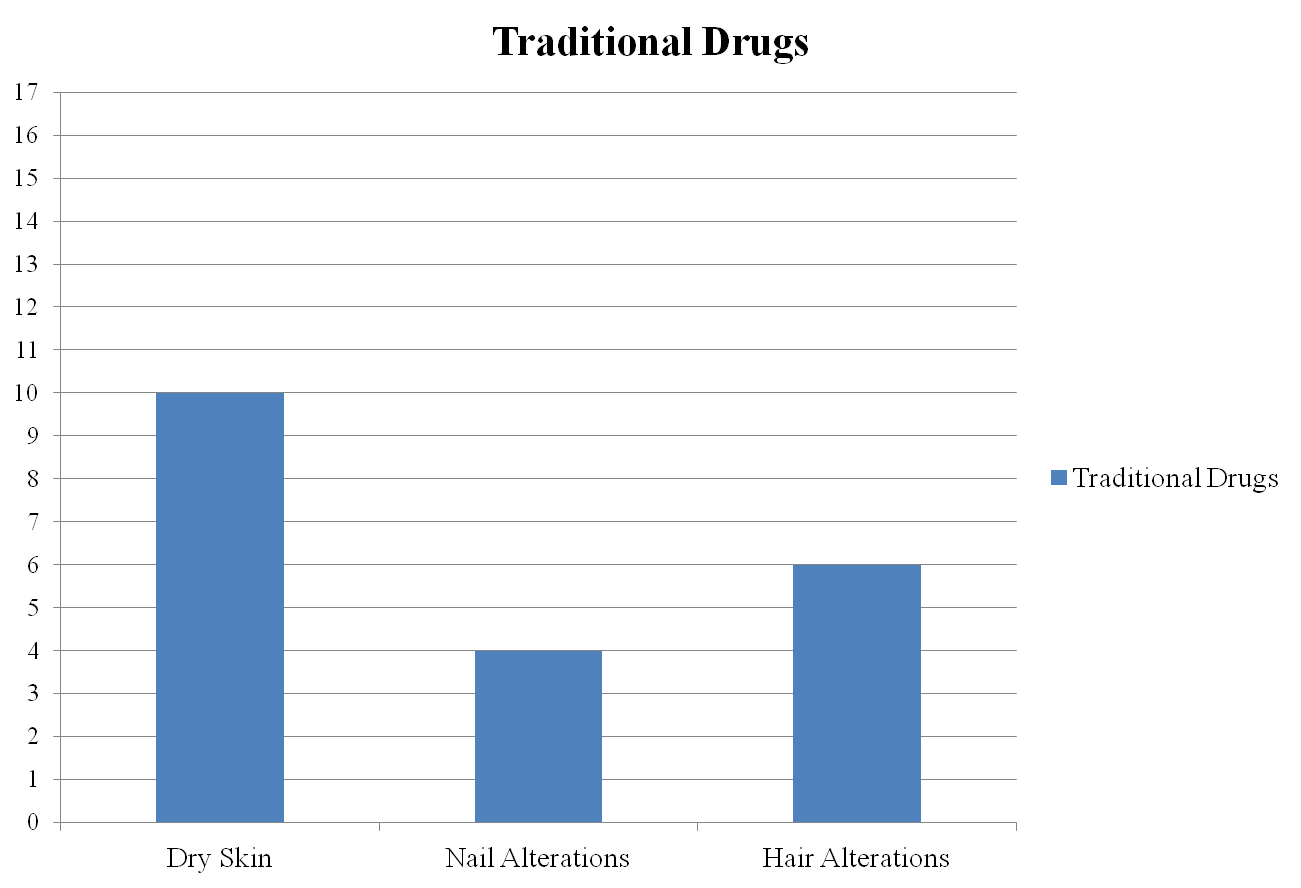

Supplement: Additional file 4 — Traditional drugs skin toxicities. [file 1756-9966-31-50-S4.png]
